# The Regenerative Potential of Decellularized Dental Pulp Extracellular Matrix: A Systematic Review

**DOI:** 10.3390/ma15186386

**Published:** 2022-09-14

**Authors:** Necdet Adanir, Zohaib Khurshid, Jithendra Ratnayake

**Affiliations:** 1 Department of Restorative Dentistry, College of Dentistry, King Faisal University, Al-Ahsa 31982, Saudi Arabia; 2 Department of Prosthodontics and Dental Implantology, College of Dentistry, King Faisal University, Al-Ahsa 31982, Saudi Arabia; 3 Faculty of Dentistry, Sir John Walsh Research Institute, University of Otago, P.O. Box 56, Dunedin 9054, New Zealand

**Keywords:** extracellular matrix, dental pulp tissue, pulp regeneration, tissue engineering, endodontics

## Abstract

Introduction: The regeneration of dental pulp remains a challenge. Although several treatment modalities have been proposed to promote pulpal regeneration, these treatments have several drawbacks. More recently, decellularized dental pulp extracellular matrix (DP-ECM) has been proposed to regenerate dental pulp. However, to date, no systematic review has summarized the overall outcome and assessed the available literature focusing on the endodontic use of DP-ECM. The aim of this systematic review is to critically appraise the literature, summarize the overall outcomes, and provide clinical recommendations about DP-ECM. Methodology: Following the Participants Intervention Control and Outcomes (PICO) principle, a focused question was constructed before conducting a search of the literature and of electronic research databases and registers. The focused question was: ‘Compared to controls, does decellularized dental pulp extracellular matrix (DP-ECM) stimulate the regeneration of dental pulp cells and tissue?’ Quality assessment of the studies was carried out using Guidelines for Reporting Pre-Clinical in Vitro Studies on Dental Materials and ARRIVE guidelines. Results: 12 studies were included in this review. Data from five in vitro experiments and eight in vivo experiments were extracted and the quality of the experiments was assessed. In majority of the studies, DP-ECM appeared to have stimulated pulpal regeneration. However, several sources of bias and methodological deficiencies were found during the quality assessment. Conclusion: Within the limitations of this review and the included studies, it may be concluded that there is insufficient evidence to deduce the overall efficacy of DP-ECM for pulpal regeneration. More research, clinical and pre-clinical, is required for more conclusive evidence.

## 1. Introduction

Dental pulp will invariably become inflamed due to infection or injury resulting from caries and trauma. The extent of the infection, and consequently, the severity of the inflammation, should dictate the proper treatment. Therefore, eliminating pulpal inflammation and/or infection is the primary objective of endodontic therapy. However, the regenerative capacity of dental pulp reduces with age due to continued deposition of secondary dentine and calcification of the pulp itself [1]. Furthermore, cellular senescence also plays an important role in reducing the regeneration of dental pulp. Therefore, endodontics in adult patients primarily involves the removal of irreversibly inflamed pulp, followed by cleaning, shaping, and obturation of the pulp chamber and the root canal. In immature and developing teeth, partial removal of dental pulp and vital pulp therapy are more effective due to the higher regenerative abilities of the pulpal tissues [2]. However, irreversible pulpitis warrants pulpal removal and apical closure, even in young permanent teeth [3]. A large proportion of teeth fail and fracture due to loss of tooth structure [4]. Therefore, contemporary materials and techniques aim to regenerate dental pulp and encourage continued development of the root rather than eradication of the dental pulp.

Decellularized extracellular matrix (dECM) has been used for the regeneration of organs such as the liver [5], heart, and nerves [6]. Decellularization of ECM leaves behind a porous, nanofibrous scaffold to support the attachment, proliferation, and infiltration of stem cells [7]. Healthy dental pulp removed from healthy teeth, extracted due to procedures such as orthodontic treatment, may be a source of ECM. Collagen and other regenerative factors present in the ECM have been observed to promote angiogenesis, which is vital for the formation of dental pulp [8]. In vitro observations indicate that DP-ECM promotes the expression of angiogenic biomarkers such as osteocalcin M (OSM) and vascular endothelial growth factor (VEGF) [7], both of which are indicative of the regeneration of blood vessels. Another advantage of a decellularized ECM is diminished antigenicity of a xenograft scaffold while keeping the nanofibrous structure intact [9]. Pulp-derived ECM has been found to contain high concentrations of glycosaminoglycans, proteoglycans, and hyaluronan, held together by a network of fibronectin and collagen-1, both of which are important to support cellular growth [10]. Recently, decellularized ECM has been studied for its potential as a regenerative scaffold for tissue engineering [11,12]. Both human and animal dental pulp can be decellularized via various protocols. Generally, the chelating agent ethylenediaminetetraacetic acid (EDTA), enzymes such as trypsin, and a lysing agent such as Triton X-100 are used to treat the pulp and separate the cellular component from the pulp to leave behind an acellular ECM [13]. The acellular dental pulp extracellular matrix (DP-ECM) may then be used for regenerative endodontics and can also be recellularized to deliver stem cells into the root canal for regeneration of the dental pulp [14]. Both in vitro and animal (in vivo) studies have suggested that DP-ECM may be used as a regenerative material to the promote regeneration of dental pulp [11,13,14]. Nevertheless, to the best of the authors’ knowledge, until now no systematic review has attempted to critically appraise and summarize the evidence focusing on the regenerative potential of DP-ECM. Therefore, the aim of this systematic review is to summarize these studies and their outcomes. Moreover, the studies will be critically appraised to evaluate their overall quality.

## 2. Materials and Methods

### 2.1. Focused Question

Following the Participants Intervention Control and Outcomes (PICO) principle [15], a focused question was constructed before conducting the literature search made according to PRISMA statement. The focused question was: ‘Compared to non-DP-ECM controls, does decellularized dental pulp extracellular matrix (DP-ECM) stimulate the regeneration of dental pulp cells and tissue?’

### 2.2. Eligibility Criteria

The following categories of articles were included: Methodology focusing on using DP-ECM (animal or human) to regenerate pulp tissues or cells and non-DP-ECM materials as controls, original articles (animal, clinical, or in vitro), and case reports/series. Articles in languages other than English, letters to the editor, and all types of reviews and commentaries were excluded.

### 2.3. Search of the Literature

**Figure 1:**
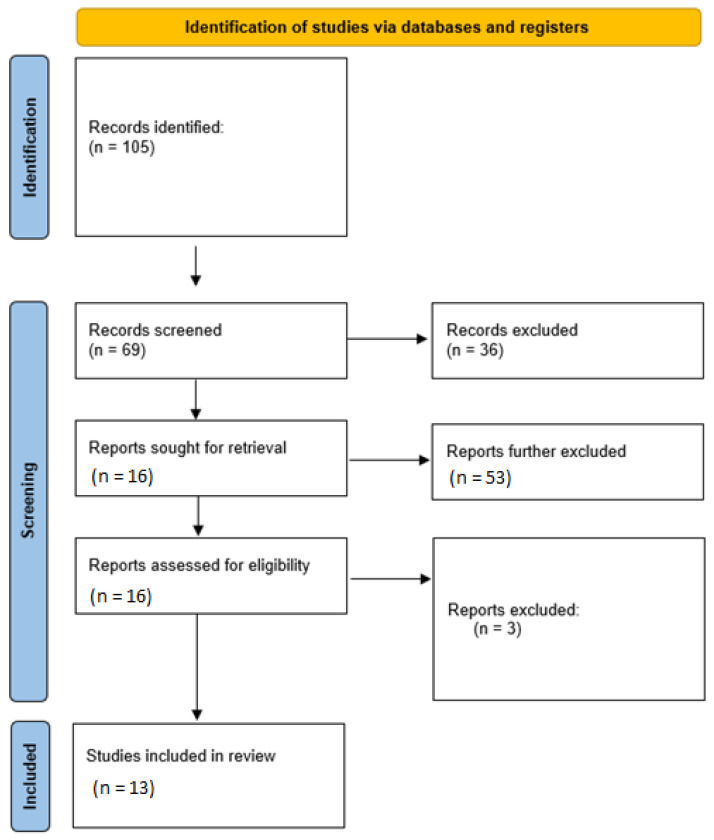
PRISMA flow diagram of the literature search employed for this review.

An electronic search was conducted via the following scientific databases: PubMED, ISI Web of Science, Google Scholar, and EMBASE [16]. The medical subject heading (MeSH) keywords used were: ‘((Dental Pulp) AND ((decellularized matrix) OR (extracellular matrix) OR (decellularized)) AND ((regeneration) OR (proliferation) OR (growth)) AND ((root canal) OR (endodontics)) OR (stem cells)).’ Filters applied were original studies and studies published until date of literature search (June 2022). Following the completion of the primary search, articles not meeting the eligibility criteria were excluded based on titles and abstracts. Potentially eligible articles were downloaded and were read comprehensively to determine their levels of inclusion. Furthermore, the reference lists of the included articles were also read to find any additional articles suitable for inclusion. A hand-search was conducted of the following journals: Journal of Endodontics, International Endodontic Journal, Dental Traumatology, and Journal of Dental Research. Moreover, a search was also conducted to find any relevant data presented in relevant conferences. All searches were conducted independently by two investigators (N.A. & Z.K.). Any disagreements were solved by discussion. The inter-examiner reliability score (κ) was calculated to quantify the degree of consistency of the articles retrieved by the two investigators. The literature search process is provided in Figure 1. Additional supplementary file number 1 is available for readers in accordance to the PRISMA statement.

### 2.4. Data Extraction

Data was primarily extracted using the PICO protocol (Participants: patients (for clinical studies)/animals (for in vivo studies)/cell cultures (for in vitro) studies; Intervention: DP-ECM; Controls: no treatment/other regenerative materials; Outcomes: regeneration of dental pulp tissues and/or cells). Data relevant to methodology, sample size, duration of the studies, and the investigations carried out were extracted from each study. Results from the cell (in vitro) studies and animal (in vivo) studies were tabulated in two different tables using predetermined data collection forms by the two investigators independently. Any disagreements were solved by discussion.

### 2.5. Quality Assessment of Studies

Depending on the type, each study was assessed individually and independently by both investigators. It was decided that for the quality assessment of any randomized clinical trials, Consolidated Standards of Reporting Trials (CONSORT) [17] would be used. The Animal Research: Reporting of In Vivo Experiments (ARRIVE) [18] guidelines were selected for animal studies and, for in vitro studies, the Guidelines for Reporting Pre-Clinical In Vitro Studies On Dental Materials [19] were used. Any disagreements were solved by discussion.

## 3. Results

### 3.1. Literature Search Results

The initial search resulted in 105 items. 36 articles were excluded based on abstracts and titles. Therefore, 69 articles were deemed potentially eligible for inclusion. A further 53 articles were excluded because they did not describe using DP-ECM for pulpal regeneration. Therefore, the full texts of 16 articles were downloaded. Three articles were further excluded because they were literature reviews. Hence, 13 articles were included in this study [7,11,13,14,20,21,22,23,24,25,26,27,28]. No additional studies were found in the grey literature or in the reference lists of the included articles. In the twelve included studies, five in vitro investigations were described [14,23,24,25,26], eight in vivo (animal) experiments were conducted [7,11,13,14,23,24,25,26,27], and, in one study, an ex vivo study model was used [28]. The overall inter-examiner reliability score (Kappa) of the literature search was calculated as 0.85.

### 3.2. General Characteristics and Overall Outcomes of In Vitro and Ex Vivo Studies

The general characteristics of the in vitro and ex vivo studies are presented in Table 1, and the animal experiment characteristics are presented in Table 2. Only one study stated the sample size in the in vitro experiments, which was 12 cell cultures [20]. In the study by Matoug-Elwerfelli et al. (2017) [20], the in vitro efficacy of collagen and DP-ECM was compared to controls (cyanoacrylate) when applied to human dental pulp tissue [23]. In another study (Song et al. [21]), three different protocols were used to decellularize dental pulp to produce DP-ECM, and their efficacy for inducing the proliferation of Stem Cells from the Apical Papilla (SCAP) was compared to that of the culture medium only. In another study, human dental pulp cells (HDP cells) were seeded in DP-ECM, collagen, and culture medium [22]. Bakhtiar et al. (2020) compared the efficacy of DP-ECM as a growth medium and culture medium using human bone marrow mesenchymal stem cells (HBMMSCs) [11]. Human dental pulp stem cells (HDPSCs) were cultured in DP-ECM and compared to those cultured in culture medium in one study [23]. In the ex vivo study by Matoug-Elwerfelli et al. (2020), rat DP-ECM was able to support the regeneration of human dental pulp tissue [28]. The duration of the experiments ranged from 7 to 14 days [11,20,21,22,23,28]. The various histological assessments are presented in Table 1.

In two studies, no difference between the outcomes in the experimental groups was observed [11,20]. In other studies, DP-ECM induced a higher differentiation of odontoblasts [22] and more proliferation of SCAP [21] compared to controls. In one study, DP-ECM induced a higher proliferation of HDPSCs and angiogenesis compared to controls [23].

### 3.3. General Characteristics and Overall Outcomes of Animal Studies

In three animal studies, rats were used [11,13,26], and mice were also used in the same number of studies [7,23,27]. Pigs were used in two studies [24,25] and beagle dogs were used in one study [14]. In three studies, the source of the DP-ECM were pigs [14,24,25], human DP-ECM was used in three studies [23,27,28], and in one study, bovine DP-ECM was used [13]. In five studies, DP-ECM was subcutaneously transplanted [13,14,23,25,27,28], and DP-ECM was delivered via electrospun scaffolds and placed in transplanted roots [24]. In one study, DP-ECM was implanted in bone defects [26], and in another study, DP-ECM was placed endodontically after removal of the pulp [14]. The duration of the in vivo experiments ranged between 1 week/7 days to 9 weeks [11,13,14,23,24,25,26,27,28]. The general outcomes, including experimental groups and the investigations carried out, are provided in Table 2. In seven animal studies, DP-ECM increased odontogenesis and angiogenesis [13,14,23,26,27,28]. In one study an increased bone regeneration was observed in the DP-ECM group [26], and in another study, DP-ECM increased pulpal regeneration in transplanted teeth [25].

### 3.4. Results of Quality Assessment of In Vitro and Ex Vivo Studies

Overall, three studies received a quality grade of ‘medium’ [11,21,23], two studies received grades of ‘low’ [20], and only study was graded as ‘high’ [11]. The assessment criteria and their results are listed in Table 3.

### 3.5. Results of Quality Assessment of Animal Studies

Type of study (animal study) was identified by two studies [23,27]. In all studies, the abstracts were adequate [11,13,14,23,24,25,26,27,28]. The rationales for the studies were described in all studies [11,13,14,23,24,25,26,27,28], but in one study, the hypothesis was not provided [24]. In three studies, the ethical statement was not provided [11,13,26]. Blinding was carried out in only two studies [11,13]. Animal groups were adequately described in six studies [11,13,14,25,26,27]. In one study, experimental procedures were not described adequately [27]. In five studies, animal test and control groups were sufficiently described [11,20,21,22,29]. None of the studies provided the details of animal housing, and none of them included a precalculated sample size [11,13,14,23,24,25,26,27,28,29]. The randomization of animal and histological samples were provided in only one study [23], and in three studies, randomization of only histological samples was provided [11,13,27]. Experimental outcomes, baseline data, and statistical calculations were provided sufficiently in all studies [11,13,14,23,24,25,26,27,28]. The numbers of experimental or control groups were provided in only one study [14]. Although outcomes were reported satisfactorily in all studies, adverse events or effects were reported in none of the studies [11,13,14,23,24,25,26,27,28]. Results were adequately interpreted in the discussion of all studies [11,13,14,23,24,25,26,27,28]. In six studies, the clinical implications of experimental results were stated [11,13,14,23,25,26], and in one study, funding information was not provided [26]. Overall, one animal study was graded as having a ‘high quality’ [23], and seven studies were given an overall grade of ‘medium’ [11,13,14,24,25,26,27,28]. The assessment criteria and their results are listed in Table 4.

## 4. Discussion

Overall, the results of this systematic review indicate that decellularized dental pulp ECM is successful in promoting the regeneration of dental pulp [29]. Nevertheless, a drawback of decellularization is the use of different reagents and enzymes, which may not only degrade the intact fibrillar network but may remain in the scaffold as potential toxins [30]. Nevertheless, the decellularization methods reported have been known to reduce DNA content equal to or less than 50 ng/mg, which is acceptable in terms of the antigenicity of the scaffolds [31].

In addition to acceptable biological properties, handling properties of scaffolds should be optimal when being applied clinically [32]. Periodontal scaffolds, such as enamel matrix derivatives (EMD), have been developed with the aim of ease of application, in addition to having regenerative properties [33]. Furthermore, another vital property of scaffolds is space maintenance, which enables tissue and cells to infiltrate into the fibrous network and pores [34]. To date, research has not been carried out to investigate these properties of DP-ECM. Additionally, no study has assessed the clinical efficacy of DP-ECM. Therefore, future studies should focus on not only optimizing and assessing the regenerative potential of the scaffold, but also work towards using DP-ECM in clinical trials. Another potential aspect of study would be a comparison between the mechanisms and efficacy of human-derived DP-ECM and xenogenic DP-ECM. Animal studies reviewed in this systematic review indicate that DP-ECM promotes angiogenesis and regeneration of pulpal tissues [13,14,23,26,27,28]. However, none of these studies compared the efficacy of the DP-ECM of currently used treatments such as autologous plasma, EMD, and even mineral trioxide aggregate [13,14,23,26,27,28] (all of which have been used clinically). Therefore, future animal studies should compare the in vivo efficacy of DP-EMD with the aforementioned materials and techniques before being permitted in the clinics.

Another avenue of interest of dental pulpal regeneration is the regeneration potential of adding exogenous growth factors to DP-ECM [35]. Indeed, in the study by Tan et al., the addition of BMP-4 potentiated the regenerative effect of DP-ECM on dental pulp [7]. Therefore, more studies should investigate this potentially viable option for pulp regeneration. To date, no studies have compared the difference in the composition and action of dental pulp extracellular matrix with those of non-dental origin. Further, it would be interesting to compare the regenerative effect of DP-ECM with other treatment options such as platelet-rich fibrin and plasma. As observed in several previous studies, the regenerative potential of the dental pulp reduces with age [36]. Since the major source of DP-ECM would be autogenous, allogenic, or xenogenic decellularized dental pulp, it is essential to choose the optimal source of DP-ECM. Although autogenous, allogenic, and xenogenic grafts for other tissues have been compared [37], to date no studies have attempted to do something similar for pulpal regeneration. The most obvious source of autologous dental pulp ECM would be decellularized dental pulp obtained from third molars or those teeth selected for pre-orthodontic extractions. However, using this option in every case would not be possible, and a xenogenic source of DP-ECM would be more logical.

In the studies reviewed, there were several limitations that may have favored outcomes. For instance, in the in vitro studies, there was significant methodological heterogeneity due to differences in histological assessments, measurements of outcomes, and duration of the experiments [13,20,21,22,23]. Therefore, with the evidence currently available from in vitro and in vivo research, the overall effect summary of DP-ECM on pulpal tissues cannot be concluded. In addition to the above-mentioned limitations of the studies included, there were several deficiencies found during the quality assessment. We discovered that only three studies employed some form of randomization [11,21,23]. A lack of randomization may have influenced the direction of results due to examiner bias. Additionally, the duration of the studies ranged from one week to nine weeks [11,13,14,23,24,25,26,27,28], which is insufficient to determine the long-term efficacy of the scaffolds for pulpal regeneration. Since dental infections involve microbial and physio-pathological etiological factors, it is imperative to carry out pulpal regenerative experimental studies on study models that simulate the microbial infective and oral microenvironments. However, none of these studies attempted to study the efficacy of DP-ECM with infected canals. Therefore, future clinical and preclinical studies should include animal models with infected canals and focus on the resolution of symptoms and signs of periapical infections, in addition to the regeneration of pulpal tissue. Due to the heterogeneity in the methodology, measurements, and results, it was not possible to conduct a meta-analysis in this systematic review. This was perhaps the most significant limitation of this review, since the mean overall efficacy of DP-ECM could not be estimated.

## 5. Conclusions

Dental pulp is a specialized dental tissue that comprises a defense system, repair and regeneration potential, sensory function cells, and resident cells. It is envisioned that a decellularized biocompatible biological scaffold containing the natural ECM structural elements necessary for tissue-specific regeneration might be created. It is possible to draw the conclusion that there are inadequate data to determine the overall effectiveness of DP-ECM for pulpal regeneration under the constraints of this review and the included research. For more clear proof, clinical and preclinical studies are still required.

## Figures and Tables

**Table 1:** General characteristics and outcomes of in vitro and ex-vivo studies included in this review.

Study (Authors, Year)	Methodology	Sample Size (*n*)	Study Groups	Duration of Experiment(s)	Investigation(s)	Outcomes
Matoug-Elwerfelli et al., 2017 [20]	HDP-ECM applied on human dental pulp tissue	*N* = 12	Cyanoacrylate glue (*n* = 4); Collagen gel (*n* = 4); ECM (*n = 4)*	14 days	DNA quantification; IHC (nucleic acids, acidic polysaccharides and collagen); cell viability and toxicity assays	No difference between control and test groups.
Song et al., 2017 [21]	SCAP seeded in HDP-ECM produced by three different methods	Not stated	Protocol 1 Protocol 2 Protocol 3 Control	2 weeks	Western blot; SEM; IHC; cell viability; Rt-PCR	HDP ECM increased proliferation of SCAP compared to controls.
Li et al., 2020 [22]	HDP cells seeded in HDP-ECM gel	Not stated	HDP cells in HDP-ECM gel HDP cells in collagen gel HDP cells in culture medium only	7 days	Cell adhesion, migration, and proliferation; Odontoblastic differentiation; IHC; Western blotting; Rt-PCR	HDP-ECM gel promoted odontoblastic differentiation.
Bakhtiar et al., 2020 [11]	hBMMSC seeded in crosslinked bovine DP-ECM	Not stated	hBMMSCs in crosslinked and non-crosslinked Bovine DP-ECM hBMMSCs in culture medium only	21 days	Proliferation and attachment assays; Rt-PCR	No statistical difference between groups.
Matoug-Elwerfelli et al., 2020 [28]	Rat DP-ECM recullarized with HDP cells.	*n* = 8	Rat DP-HDP seeded with HDP cells (*n* = 4) Cellular rat dental pulp seeded with HDP (*n* = 4)	14 days	Biocompatibility, LIVE/DEAD assay, immunohistology, odontoblast differentiation	Rat DP-ECM was able to support human pulp regeneration
Alghutaimel et al., 2021 [23]	HDPSCs cultured in DP-ECM	Not stated	HDPSCs + DP-ECM HDPSCs + Culture medium	7 days	IHC (collagen type I, dentin matrix protein 1, dentin sialoprotein, and Von Willebrand); ELISA (transforming growth factor β, vascular endothelial growth factor, and basic fibroblast growth factor)	Increased proliferation and angiogenic factor expression when HDPSCs cultured in DP-ECM compared to medium only.

SCAP: stem cells from the apical papilla; hHBMMSCs, human bone marrow mesenchymal stem cells; HDPSCs, human dental pulp stem cells.

**Table 2:** General characteristics and outcomes of the in vivo studies included in this review.

No.	Study (Author, Year)	Animal Model (*n*)	Source of DP-ECM	Methodology	Study Groups (*n*)	Duration	Investigations	Outcomes
1	Chen et al., 2015 [24]	Pig (*n* not stated)	Pig	Scaffold/TDM/DP-ECM/Scaffold + TDM + DP-ECM placed in transplanted roots	E-spun scaffolds Treated dentine matrix DP-ECM Scaffold + TDM + DP-ECM	7 days	SEM; IHC; Histology	Scaffold + TDM + DP-ECM promoted regeneration of root and dental pulp tissues.
2	Hu et al., 2017 [25]	Pigs (*n* = 9); Immunodeficient mice (*n* not stated)	Pig	Seeding human dental pulp stem cells into swine decellularized pulp and transplanted subcutaneously into nude mice	Not stated	8 weeks	SEM; H&E staining; IHC;	DP-ECM promoted pulpal regeneration in transplanted teeth.
3	Alqahtani et al., 2018 [14]	Beagle dogs (*n* = 2)	Pig	Porcine DP-ECM, collagen and blood clot alone compared with each other for pulpal regeneration in the root canal.	DP-ECM (*n* = 2 teeth) Collagen (*n* = 3 teeth) Blood clot (*n* = 3 teeth)	8 weeks	Micro-CT; IHC (CD31 and DSP)	DP-ECM promoted pulp regeneration and angiogenesis more than collagen and blood clot.
4	Bakhtiar et al., 2020 [11]	Sprague Dawley rats (*n* = 24)	Bovine	Crosslinked and non-crosslinked bovine DP-ECM implanted subcutaneously.	Crosslinked Bovine DP-ECM - 1.5 mg/mL - 2.25 mg/mL - 3.00 mg/mL Non-crosslinkedBovine DP-ECM - 1.5 mg/mL - 2.25 mg/mL - 3.00 mg/mL *n* for each group not stated	2 weeks	Histology and IHC	Cross-linked scaffolds degraded at a lower rate but produced lesser inflammation compared to non-crosslinked scaffolds. More angiogenesis observed in crosslinked group.
5	Lee et al., 2020 [26]	Rats (*n* = 6)	Human	HDP-ECM with and without BMMSCs implant into defects in calvaria	HDP-ECM only HDP-ECM + BMMSC	12 weeks	Micro-CT and histology	Angiogenesis and bone formation observed in both groups
6	Alghutaimel et al., 2021 [23]	Immunodeficient mice (*n* not stated)	Human	HPDP-ECM with and without HDPSCs implanted subcutaneously.	HDP-ECM + HDPSCs HDP-ECM only No treatment *n* for each group not stated	30 days	IHC; Histology	HDP-ECM + HDPSCs promoted the highest amount of angiogenesis
7	Bakhtiar et al., 2021 [13]	Sprague Dawley (*n* not stated)	Bovine	Bovine DP-ECM implanted subcutaneously.	Bovine DP-ECM	2 weeks	Immune response	Immune response followed by angiogenesis and fibrous encapsulation.
8	Kim et al., 2021 [27]	Immunodeficient mice (*n* = 20)	Human	Human PDLSCs and DPSCs seeded on human DP-ECM transplanted subcutaneously.	HPDL-ECM + PDLSCs HDP-ECM + DPSCs HPDL-ECM HDP-ECM *n* for each group not stated.	9 weeks	Histology; IHC	Pro-angiogenic and regenerative biomarkers detected.
9	Tan et al., 2021 [7]	Mice (*n* not stated)	Human	Human DPSCs and BMP-4 (via recombinant adenovirus) seeded on human DP-ECM transplanted subcutaneously.	PBS + DPSCs GFP + DPSCs DP-ECM + DPSCs BMP4 + DPSCs BMP4 + DP-ECM + DPSCs	4 weeks	Gene expression; histology; IHC	BMP-4 promoted upregulation of the expression of osteogenic, odontogenic and angiogenic markers in DPSCs seeded on DP-ECM.

DSP, dentin sialoprotein; DP-ECM, dental pulp extracellular matrix; BMMSCs, bone marrow mesenchymal stem cells; BMP-4 bone morphogenic protein-4; GFP, green fluorescent protein; ECM, extracellular matrix.

**Table 3:** Results of the quality assessment conducted on the in vitro and ex vivo studies or experiments included in this review.

Assessment Item	Matoug-Elwerfelli et al., 2017	Song et al., 2017	Li et al., 2020	Bakhtiar et al., 2020	Matoug-Elwerfelli et al., 2020	Alghutaimel et al., 2021
1. Introduction						
(a) Objectives	Yes	Yes	Yes	Yes	Yes	Yes
(b) Methods	Yes	Yes	Yes	Yes	Yes	Yes
2. Replicable methods	Yes	Yes	Yes	Yes	Yes	Yes
3. Adequate outcomes	Yes	Yes	Yes	Yes	Yes	Yes
4. Predetermined sample size	No	No	No	No	No	No
5. Allocation of samples	No	No	No	No	No	No
6. Randomization						
(a) Allocation concealment	No	No	No	Yes	No	No
(b) Implementation	No	Yes	No	Yes	No	Yes
(c) Blinding	No	No	No	Yes	No	No
7. Statistics	Yes	Yes	Yes	Yes	Yes	Yes
8. Adequate outcomes & estimation	Yes	Yes	Yes	Yes	Yes	Yes
9. Discussion: Limitations	No	No	No	No	No	No
10. Funding	No	No	Yes	Yes	No	Yes
11. Accessible protocol	No	No	No	No	No	No
Overall quality	Low	Medium	Medium	High	Low	Medium

**Table 4:** Results of the quality assessments of the included animal studies.

Study Characteristics	Chen et al., 2015	Hu et al., 2017	Alqahtani et al., 2018	Bakhtiar et al., 2020	Lee et al., 2020	Alghutaimel et al., 2021	Bakhtiar et al., 2021	Kim et al., 2021	Tan et al., 2021
**Animal study identified in title**	No	No	No	No	No	Yes	No	Yes	No
**Abstract**	Yes	Yes	Yes	Yes	Yes	Yes	Yes	Yes	Yes
**Introduction**									
Adequate background	Yes	Yes	Yes	Yes	Yes	Yes	Yes	Yes	Yes
Objectives/hypotheses described adequately	No	Yes	Yes	Yes	Yes	Yes	Yes	Yes	Yes
**Method**									
Ethical statement	Yes	Yes	Yes	No	No	Yes	No	Yes	Yes
Blinding	No	No	No	Yes	No	No	Yes	No	No
Description of animal groups	No	Yes	Yes	Yes	Yes	No	Yes	No	Yes
Adequate experimental procedures	Yes	Yes	Yes	Yes	Yes	Yes	Yes	No	Yes
Experimental animal groups and controls	Yes	Yes	Yes	No	No	Yes	No	No	Yes
Housing details	No	No	No	No	No	No	No	No	No
Precalculated sample size	No	No	No	No	No	No	No	No	No
Randomization of teeth/animals	No	No	No	Only histological samples	No	Yes	Only histological samples	Only histological samples	No
Experimental outcomes	Yes	Yes	Yes	Yes	Yes	Yes	Yes	Yes	Yes
Statistics	Yes	Yes	Yes	Yes	Yes	Yes	Yes	Yes	Yes
**Results**									
Baseline data	Yes	Yes	Yes	Yes	Yes	Yes	Yes	Yes	Yes
Number analyzed/animals lost	No	No	Yes	No	No	No	No	No	No
Adequate outcomes	Yes	Yes	Yes	Yes	Yes	Yes	Yes	Yes	Yes
Reporting of adverse effects	No	No	No	No	No	No	No	No	No
**Discussion**									
Adequate interpretation of results	Yes	Yes	Yes	Yes	Yes	Yes	Yes	Yes	Yes
Clinical implications	No	Yes	Yes	Yes	Yes	Yes	Yes	No	No
**Funding information**	Yes	Yes	Yes	Yes	No	Yes	Yes	Yes	Yes
**Overall quality**	Medium	Medium	Medium	Medium	Medium	High	Medium	Medium	Medium

## Data Availability

Not applicable.
